# Resistin and Visfatin Expression in HCT-116 Colorectal Cancer Cell Line

**Published:** 2013

**Authors:** Sara Ghaemmaghami, Seyed Mojtaba Mohaddes, Mehdi Hedayati, Masumeh Gorgian Mohammadi, Golnoosh Dehbashi

**Affiliations:** 1 Department of Clinical Biochemistry, Faculty of Medicine, Tabriz University of Medical Sciences, Tabriz, Iran.; 2 Department* of Medical* Genetics, Faculty of Medicine, Tabriz University of Medical Sciences, Tabriz, Iran.; 3 *Cellular and Molecular Endocrine Research Center, Research Institute for Endocrine Sciences, Shahid Beheshti University of Medical Sciences, Tehran, Iran.*

**Keywords:** Visfatin, resistin, colorectal cancer

## Abstract

Adipocytokines, hormones secreted from adipose tissue, have been shown to be associated with many cancers such as breast, prostate and colorectal cancer. Recent studies have indicated that resistin and visfatin, two of these adipokines have high level plasma concentrations in colorectal cancer patients and may be promising biomarkers for colorectal cancer. The aim of this study was to identify whether the colorectal cancer cell line, HCT-116, itself is the source of these two adipokines secretion. Resistin and visfatin expression were investigated in HCT-116 by RT – PCR at mRNA level and confirmed by ELISA at protein level. Visfatin showed a high expression at both mRNA and protein levels in HCT-116. Conversely, resistin was not expressed in either cell lysate or supernatant. These results showed that HCT-116 colorectal cancer cells secrete and express visfatin endogenously. However, they are not the main source of resistin and the high level of resistin in colorectal cancer may be due to monocytes and other inflammatory cells which increase in proinflammation status of cancer. Taken together, visfatin may act on colorectal cancer cell in an autocrine manner while resistin may act in a paracrine manner.

Despite previous beliefs that fat tissue is a passive organ for storing excess calories, today it is known as a dynamic endocrine organ with high metabolic activity which synthesizes and secretes a large number of biologically active molecules called adipokines including leptin, adiponectin, TNF-α, IL-6 and resistin (1). In addition to adipocytes, the main source of adipokines, other cells and tissues can produce some of them. For example, Leptin can also be synthesized by bone marrow, syncytiotrophoblasts of placenta, human mammary epithelial cells, gastric and colonic inflamed epithelial cells (2-4). Adiponectin is produced by placenta and skeletal muscles as well as adipose tissue (5-6). Previous studies have indicated the association between adipokines and many obesity-related disorders including malignancy (7-8). Leptin serum level is increased in prostate and colorectal cancer (CRC) and plays a key role in hepatocellular carcinoma and breast cancer (9-12). Recent study has revealed that resistin and visfatin plasma levels are significantly higher in colorectal cancer patients than control group and thus may be good biomarkers for colorectal cancer (13).

Resistin, also named ADSF (adipocyte secreted factor) or FIZZ-3 (found in inflammatory zone) is a member of RELMs (resistin-like molecules) family protein with cysteine rich structure. It is identified as a 12.5 kDa polypeptide related to human chromosome 19 (14, 15). Resistin has been shown to play an important regulatory role in adipogenesis, glucose hemostasis and insulin sensitivity. It is also expressed in the hypothalamus and can activate hypothalamic neurons to control food intake (14).

Resistin plasma level is increased in many inflammation related disorders such as atherosclerosis, chronic inflammatory bowel disease, chronic renal disease, systemic lupus erythematosus (SLE), arthritis (14, 16-19). Moreover, high serum level of resistin has been found in many tumors including breast, prostate and colorectal cancer which implies its relationship to malignancy (13, 20-21). However, the main source of resistin reflecting its increased serum levels in cancers especially in colorectal cancer is still ambiguous.

Visfatin/Nampt, another adipokine with 25 KDa polypeptide which belongs to the dimeric class of type II phosphoribosyltransferases, is a rate-limiting enzyme in NAD biosynthesis which exists as intracellular (iNampt) in the nucleus, cytoplasm and mitochondria and extracellular (eNampt) forms (22-23). Since it was initially described as a growth factor for early B cell proliferation isolated from peripheral blood lymphocytes, PBEF (pre-B- cell colony-enhancing factor) is also used to refer to this protein (24).

Visfatin has been shown to have insulin mimetic effect and plays many pathophysiologic conditions, including metabolism, immune response and cancer (25-26). Visfatin up-regulation has been reported in many inflammatory related disorders such as atherosclerosis, inflammatory bowel disease, psoriasis, rheumatoid arthritis, osteo-arthritis, acute lung injury and chronic obstructive pulmonary disease (14). There are also several documents revealing visfatin expression in many malignant tissues including prostate, breast, endometrium and glioblastoma (27-30). Although high visfatin plasma level has been found in colorectal cancer patients, the ability of colorectal cancer cell to express this adipokine and the main cause of its high plasma concentration is still unclear.

To clarify the colorectal cancer cell potential to secrete these two adipokines, we looked for resistin and visfatin mRNAs and proteins in HCT-116 cell line which is the epithelial cell derived from colorectal adenocarcinoma tissue and positive for TGF-β1 and TGF-β2 expression and has also a mutation in codon 13 of the ras protooncogene. For the current ex-vivo study, the four cell line's characterizations were important: 1) being related to colorectal cancer tissue. 2) availability. 3) being free of viruses. 4) being derived from the tumor site but not the metastatic site. HCT-116 cell lines fulfilled all the mentioned properties.

## Materials and Methods

**Cell culture**

Human HCT-116 colorectal epithelial cancer cells were bought from Pasteur Institute Cell Bank (IRI) and grown in high glucose DMEM medium (Invitrogen, USA) supplemented with 10% fetal bovine serum (Biochem, Germany), penicillin/str-eptomycin (100U/ml and 100 mg/ml, respectively) at 37˚C, under 5% CO_2_ atmosphere. The cells were seeded at 1.5×10^6^ cells in 25-T culture flasks and allowed to attach overnight in an incubator. The total medium was then replaced with serum-free medium for 24 h and 48h to be ensured about the absence of any similar factors in the medium disturbing these adipokines study in ELISA method and allow the cells to secrete fresh proteins. Since intracellular proteins are isolated from the medium and there is no confusing factor for adipokine study in cell lysate samples, serum-free medium replacement was not done for such samples. Therefore, the cells were harvested after overnight incubation to achieve the same cell counts (1.5×10^6^).

**Cell preparation and ELISA**

The supernatant of HCT-116 cells was obtained from 24 and 48 hours serum free cell flasks. After harvesting cells by trypsin-EDTA and centrifugation, 1 ml cell lysis buffer 1X( contains 20 mM Tris-HCl (Ph 7.5), 150 mM NaCl, 1 mM Na_2_EDTA, 1 mM EGTA, 1% Triton, 2.5 mM Sodium pyrophosphate, 1 mM beta-glycero-phosphate, 1mM Na_3_VO_4_, 1μg/ml leupeptin) and 10 μl problock^TM ^Gold Protease Inhibitor Cocktail (contains Dimethyl Sulfoxide 95-100%, 4-(2-Aminoethylbenzenesulfonyl fluoride hydrochlor-ide) <2% and Alpha-toluenesulfonyl fluoride <2%) from Gold biotechnology (USA) were added, then vortexed and centrifuged for 5 min at 4°C to obtain supernatants containing the cell lysate. In order to find the resistin protein, a human resistin ELISA kit based on quantitative sandwich enzyme immuno-assay technique (Koma Biothech, Korea) with a sensitivity of 0.01ng/ml an inter-assay and intra-assay coefficient of variation of less than 10% was used. Visfatin peptide was searched by using visfatin enzyme immunoassay (EIA) kit, an *in vitro* quantitative assay based on the principle of competitive enzyme immunoassay (RayBio®, USA) with sensitivity of 0.4ng/ml, an intra-assay coefficient of variation of less than 10% and an inter-assay coefficient of variation of less than 15%. Standards, controls, and samples were assessed at the wavelength of 450 nm. Triplicate measurements were performed in a single experiment.

**RNA extraction, cDNA synthesis and RT-PCR**

Total RNA was isolated from the cultured cells using RNX plus solution (Cinna Gen INC, IRI). Briefly, after harvesting the cells by trypsin/ EDTA and centrifugation at 1000 g for 10 min at 4°C, 1 ml ice cold guanidine/ phenol solution (RNX-Plus) was added to a 2ml tube containing cell pellet and incubated at room temperature for 5 mins. Then, 200 μl of chloroform was added and shaken rigorously for 15 seconds and incubated on ice for 5 mins. The mixture was centrifuged at 12000 g for 15 mins. The aqueous phase was transferred to a sterile RNase-free tube. The total RNA was precipitated by adding 0.5 ml isopropyl alcohol and incubating for 15 mins at 24°C*.* The pellet including total RNA was washed using 75% ethanol and centrifuged at 7500 g for 8 mins. After the ethanol was dried, the RNA pellet was dissolved in DEPC treated water. The RNA yield and purity were assessed by spectrophotometric analysis. To confirm the absence of RNA degradation, RNA electrophoresis was performed on a 1.5% agarose gel containing ethidium bromide.

Total RNA (3 μg) from each sample was subjected to reverse transcription (RT) by the First strand cDNA synthesis kit (Fermentas, Lithuania) according to the manufacturer’s instruction using random hexamer primers.

In order to study resistin and visfatin expression, the RT reaction aliquot was amplified several times with temperature gradient conditions in a total volume of 50 μl using resitin primers, visfatin primers and GAPDH primers, as a housekeeping gene. The sequences of primers and the product sizes are shown in table 1. Reaction products were run on polyacryl-amide gel as triplicate.

## Results

To determine the visfatin and resistin expression and secretion from colorectal cancer cells, cell lysate and supernatant of HCT-116 cells subcultured at 1.5×10 ^6^ cells in culture flasks were obtained and then examined using enzyme immunoassay (EIA) kit. The results showed that the supernatant of 1.5×10 ^6^ HCT-116 cells contained 2425 ng/ml and 2637.52ng/ml visfatin protein after 24 and 48 hours, respectively. However, resistin protein was not detected in HCT-116 cell super-natants. The cell lyaste contained 3050.4 ng/ml visfatin protein per 1.5×10 ^6^ cells, but no detectable resistin protein. All values are presented as the mean of three reaction tubes per group.

**Table.1:** sequences of primers for RT-PCR

Gene	Location	Sequence	Product size (bp)
**Visfatin**			
Forward primer Reverse primer	1442F 1630R	TGCCTTCGGTTCTGGTGGAGGTT ACAAAATTCCCTGCTGGCGTCCT	189
**Resistin**			
Forward primer Reverse primer	108F 317R	TGGAAGAAGCCATCAATGAGAGG CGCACTGGCAGTGACATGTG	210
**GAPDH**			
Forward primer Reverse primer	681F 1176R	CAAGGTCATCCATGACAACTTTG GTCCACCACCCTGTTGCTGTAG	496

**Fig. 1:**
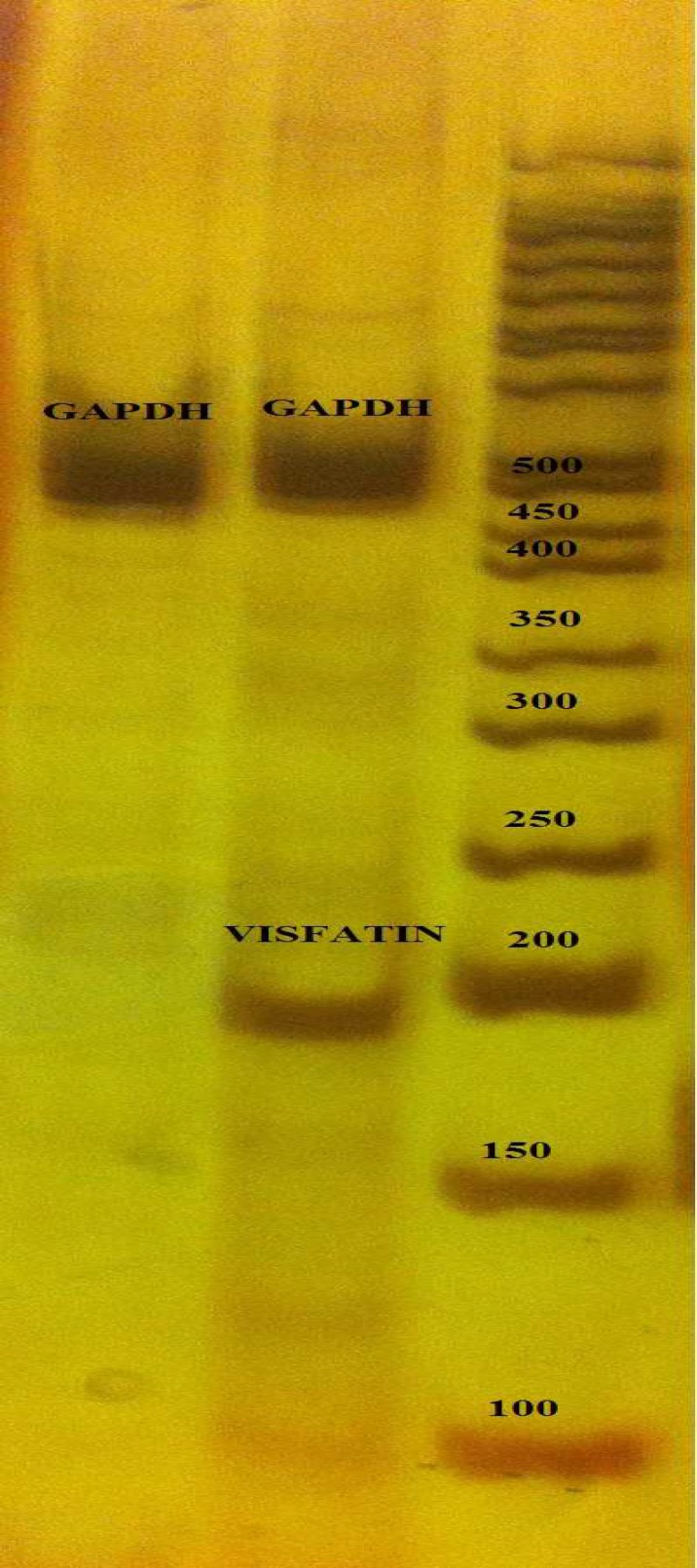
RT-PCR results of visfatin and resistin mRNA expre-ssion in HCT-116 colorectal cancer cell line

To investigate resistin and visfatin mRNAs in cancer cells, the cDNA from total RNA of HCT-116 cells was amplified with specific primers. The RT-PCR yield was followed on polyacrylamide gel by electrophoresis. As shown in figure 1, mRNA of visfatin was dominantly expressed in HCT-116 cells, while resistin mRNA was not found. RT-PCR reactions were repeated three times per group.

As the aim of this study was to find whether HCT-116 cell lines are able to express these two adipokines and not to compare its ability with the normal cells, no other cell culture was investigated for the presence of these mRNAs. Figure 1 shows the RT-PCR results of resistin and visfatin genes. Visfatin showed a sharp band at 189 bp, however, cDNA band of resistin was not detected on gel. GAPDH primers were used in RT-PCR asinternal control.

## Discussion

Despite the fact that adipose tissue is a primary source of adipokines and obesity, the condition accompanied with high serum levels of adipokines and related to many malignancies including colorectal cancer (31-34), recent investigation has demonstrated that resistin and visfatin plasma levels are significantly increased in colorectal cancer (CRC) patients independent of BMI and may be good biomarkers for malignant potential and stage progression of this cancer (13, 32). However, the other major sources of these two adipokines may also be responsible for their high serum concentrations in CRC patients rather than adipose tissue. To date, there are only a few documents about resistin and visfatin expression in CRC (15, 35-36).

A previous study has compared resistin protein expression in cancers with paired normal tissue using ELISA and western blot methods and reported an upregulation of resistin in CRC tissues. Immunohistochemistry analysis has shown a heterogeneous and diffuse weak pattern of resistin expression in cancer tissues. This expression has been referred to CD68 + cells, identified as macrophages predominantly found in cancer tissues. However, no association between resistin level in cancer tissue and plasma levels or clinical characteristics such as Dukes’ stage, location and gender has been reported (15). In this study, the colorectal cancer cell line ability was assessed to express resistin mRNA and protein. In concordance with an earlier study, our findings showed that colorectal cancer cell line HCT - 116 can not be the source of resistin production in CRC and there may be other cells in cancer stroma secreting this adipokine.

Interestingly, it has been reported that in human peripheral blood mononuclear cells (PBMCs), macrophages and bone marrow cells are the main origin of resistin production while human adipocytes can express resistin only moderately (37-38). Moreover, as mentioned earlier, resistin is linked with many chronic inflammatory conditions, including cancers (14, 16-19, 39). Resistin mRNA expression has been demonstrated to be enhanced by PBMCs stimulation with endotoxin or pro-inflammatory cytokines such as IL-1, IL-6 and TNF-α (37).

On the other hand, the association between resistin plasma level, independent from BMI, and CRP (C - reactive protein) as an inflammatory phase protein factor in inflammatory related disorders including cancers especially CRC has been widely reported (40-42). While resistin expression in prostate cancer tissue and cell lines is detectable (43), our result showed that HCT-116 colorectal cancer cell has no potential to synthesize resistin. Taken together, it seems reasonable to consider monocytes and other inflammatory cells, but not the colorectal cancer cells, as the main responsible of the high resistin expression in CRC tissue and plasma level due to the chronic low grade inflammation status in CRC. Since the receptor of resistin is still not identified, the molecular pathway of its effect on cell is not clear and deserves more investigations.

Although the visceral adipose tissue was known to produce visfatin predominantly, visceral and subcutaneous adipose tissues have not shown significant differences in visfatin mRNA expression. On the other hand, this adipokine is not only an adipocyte-specific protein. Human peri-pheral blood lymphocytes were first found to express this protein (14). Moreover, neutrophils can also produce visfatin in response to inflammatory stimuli which inhibits apoptosis in inflammation condition (44). The other sources of visfatin gene expression are the PBMCs and peripheral blood granulocytes (PBG) in synovial tissue of rheumatoid arthritis patients. Visfatin has been reported to be highly synthesized in human osteoarthritis chondrocytes (14). All these reports show the link of visfatin and inflammatory status.

As mentioned earlier, visfatin has been observed to be over expressed in many malignant tissues such as breast and prostate cancer (27-28). visfatin over expression in colorectal cancer tissue was first reported by substractive hybridization method and confirmed by RT-PCR, Northern analysis and in situ hybridization in 1999 (35). However, the cell responsible for its secretion has not been definitely identified. The subsequent study on colorectal cancer tissue using peptide-specific phage antibodies has indicated visfatin over expression in protein lysate of cancerous tissue. Immunohistochemical staining of the tumor stromal compartment did not reveal the presence of visfatin in epithelial cells (36). In contrast, our findings showed that HCT-116 colorectal cancer cell line has the ability to produce visfatin protein. The concentration of this adipokine was greater in cell lysate compared to supernatants and secretion of visfatin was increased over time. Noteworthy, there are various colorectal cancer cell lines with different characterizations presenting highly variable gene expression, growth and metastatic capacities which can result in clinically heterogeneous cancer progression (45). In the present study, only one cancer cell line was examined and therefore, in order to be explicit if there are variable visfatin expression abilities of CRC cells, further cellular investigations focusing on colorectal cancer are needed.

Nevertheless, the result of the present study showed that HCT-116 cells secrete and express visfatin protein endogenously which may apply its possible carcinogenous effects by the autocrine manner, although lymphocytes and neutrophils and other immune system cells existing in stroma compartment of cancerous tissue secrete this adipokine as an inflammatory phase protein, due to the inflammation status of colorectal cancer. Therefore, immune cells visfatin may affect colorectal cancer cells in a paracrine manner.

Taken together, it can be concluded that while visfatin protein can be synthesized by HCT-116 colorectal cancer epithelial cells, resistin, the other possible CRC biomarker is not expressed by colorectal cancer cells. Moreover, since inflamma-tion may be present to a certain extent in CRC, immune cells standing in cancerous tissue can also be the source of both visfatin and resistin expression leading to their high serum levels. Notably, although adipose tissue function role is less than others, it should still be known as the origin of these two adipokines in CRC. In other words, visfatin can affect colorectal cancer cells in an autocrine or paracrine and probably slightly in an endocrine (produced by adipocytes) manner, while resistin plays its possible carcinogenesis role in CRC only through paracrine and endocrine manner.
